# Supplementary material for: Assessing the asymmetric war-growth nexus: A case of Afghanistan
Source: PLoS One. 2022 Aug 17;17(8):e0272670. doi: 10.1371/journal.pone.0272670 (PMC9385010; doi:10.1371/journal.pone.0272670)
Supplement: Attachment — Submitted filename: Review of PONE-D-22-08275.pdf [file pone.0272670.s001.pdf]

Review of "Assessing the war-economic growth nexus: A case of Afghanistan"

Summary:

The authors empirically investigate the impact of Afghanistan's long-term war (1980 to 2020) on its economic growth. An Autoregressive Distributed Lag (ARDL) modelling approach has been applied for the data period of 2002 to 2018. The results indicate that an increase in the cost of the war increase the per capita GDP in Afghanistan for the study period, ceteris paribus. The outcomes may serve as a basis for designing policies and strategies for Afghan and international policymakers involved in Afghanistan.

Major Comments:

- Please review the writing of the paper.

- I believe that the paper is well-motivated, and the literature review is well-done.

- The ARDL econometric method is appropriate to study the hypotheses of the paper, and the method is performed well.

Minor Comments:

- Please improve the structure in Section 3.7.
