# Supplementary material for: Assessing the asymmetric war-growth nexus: A case of Afghanistan
Source: PLoS One. 2022 Aug 17;17(8):e0272670. doi: 10.1371/journal.pone.0272670 (PMC9385010; doi:10.1371/journal.pone.0272670)
Supplement: Attachment — Submitted filename: Comments.docx [file pone.0272670.s002.docx]

Comments on “Assessing the war-economic growth nexus: A case of Afghanistan” (PONE-D-22-08275)

War is an important factor for a countries’ economy, this paper considers an Afghanistan sample to show the relationship between the cost of war and per capita gross domestic product and per cost of war. They use an ARDL method to study the long- and short-term impact of war on GDP. There are still some problems to be solved in order to improve the quality of this paper, which are:

1. Why do authors consider only the Afghanistan sample, and authors should show the generality of results of this paper?

2. Author should re-organize the literatures so that show the contribution.

3. In the empirical test part, authors should explain the reason for choosing the control variables. There would be many other factors which can damage the economy, such as global financial crisis, climate change and diseases and commodity price, does the author consider these factors? Does the author consider the variation of the number of populations?

4. Authors are encosuraged to add more robust check, such as the method of costs?

5. Some related papers would be useful, see,

[1] Zhifang He, Jiaqi Chen, Fangzhao Zhou, Guoqing Zhang, Fenghua Wen. Oil price uncertainty and the risk-return relation in stock markets: Evidence from oil-importing and oil-exporting countries. Int J Fin Econ. 2020;1–19. https://doi.org/10.1002/ijfe.2206
[2] Jie Cao, Fenghua Wen, H E Stanley, Xiong Wang. Multilayer financial networks and systemic importance: Evidence from China[J]. International Review of Financial Analysis, 2021: 101882.
[3] Xin Yang, Shigang Wen, Zhifeng Liu, Cai Li, Chuangxia Huang. Dynamic Properties of Foreign Exchange Complex Network
[4] Xian Chen, Yang Li, Jihong Xiao, Fenghua Wen. Oil shocks, competition, and corporate investment: Evidence from China[J]. Energy Economics. 2020, 89: 104819. <https://doi.org/10.1016/j.eneco.2020.104819>
[5] Jie Cao, H. E. Stanley, Fenghua Wen. Measuring the systemic risk in indirect financial networks[J]. *The* *European Journal of Finance*, 2021: 1-46. <https://doi.org/10.1080/1351847X.2021.1958244>
